# 968. The Impact of Film Array Detection of High-risk and Resistant Pathogens on the Time to Optimal Antibiotic Therapy at a Community Health System

**DOI:** 10.1093/ofid/ofac492.810

**Published:** 2022-12-15

**Authors:** Tyler W Baumeister, Emily Sinclair, Dustin Zeigler, Jeremy J Frens

**Affiliations:** Cone Health, Greensboro, North Carolina; Cone Health, Greensboro, North Carolina; Cone Health, Greensboro, North Carolina; Cone Health, Greensboro, North Carolina

## Abstract

**Background:**

The importance of effective antibiotic therapy for the treatment of bloodstream infections has been well described. Rapid diagnostic testing platforms like the BioFire^®^ FilmArray^®^ blood culture identification (BCID) have the potential to decrease time to effective antibiotics when acted on appropriately. The Cone Health stewardship team has collaborated with clinical pharmacists and infectious diseases physicians to define a process to optimize antibiotic therapy. This process involves microbiology calling a pharmacist with newly positive blood cultures, and the pharmacist notifies the attending physician with the results and evidence-based treatment recommendations. We transitioned from BCID to BCID2 in August, 2021. As CTX-M (marker for extended spectrum beta-lactamase production) was not a target on BCID, we were interested in how the addition of this marker could improve care in this subset of patients.

Procedure for Positive Blood Culture Workup and Notification

**Figure:**
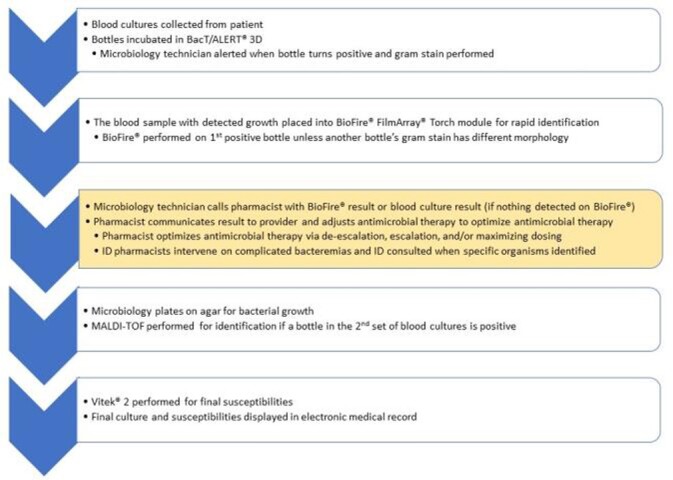

Treatment Recommendations for Selected Organisms

**Figure:**
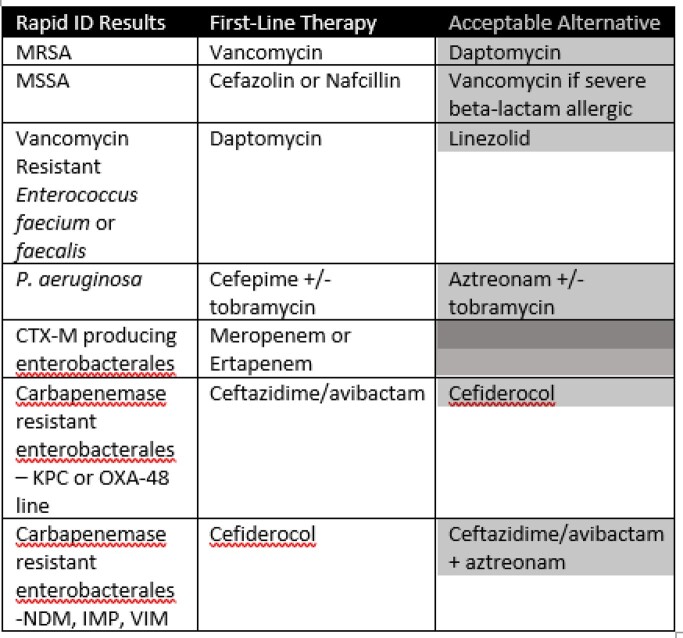

**Methods:**

From August 2021 - March 2022, the times to administration of effective and optimal antibiotics were evaluated in patients with the following BCID2 organism and resistance gene targets identified: *S. aureus, P. aeruginosa,* CTX-M, KPC, OXA-48 like, IMP, VIM, NDM, and vancomycin resistant enterococci. An effective antibiotic was defined as an antibiotic with *in vitro* activity to the organism, and an optimal antibiotic was defined as the preferred agent based on institutional guidelines. For CTX-M isolates, we compared effective and optimal times to a historical control of ceftriaxone resistant enterobacterales and analyzed by student’s t-test.

**Results:**

The combined mean time from BCID2 to administration of effective antibiotics = 1.2 hours (range 0-7.9 hours) and to optimal therapy =7.6 hours (range 0 – 113.8 hours). Prior to the BCID2 transition, patients found to be growing ceftriaxone resistant enterobacterales had an average time to optimal antibiotic administration of 17.7 hours, which improved to 2.8 hours after BCID2 implementation (p=0.0041).

Time to Effective and Optimal Antibiotics after implementation of BCID2 (August 2021 – March 2022)

**Figure:**
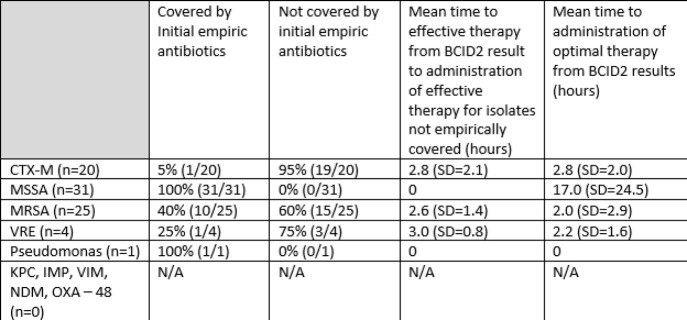

Time to Effective and Optimal Antibiotics for Ceftriaxone Resistant Enterobacterales Prior to BCID2 Implementation (January 2021 - July 2021)

**Figure:**
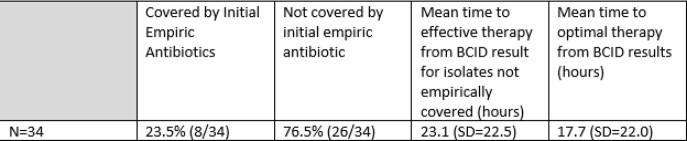

**Conclusion:**

BioFire^®^ FilmArray^®^ coupled with antibiotic stewardship team support can help optimize time to effective and optimal antibiotic therapy in bloodstream infection. The addition of the CTX-M target to BCID2 was a helpful addition to the panel which allowed for decreased time to optimal antibiotic therapy.

**Disclosures:**

**All Authors**: No reported disclosures.

